# An auto-antibody identified from phenotypic directed screening platform shows host immunity against EV-A71 infection

**DOI:** 10.1186/s12929-022-00794-2

**Published:** 2022-02-08

**Authors:** Yu-Wei Cheng, Yung-Chun Chuang, Sheng-Wen Huang, Ching-Chuan Liu, Jen-Ren Wang

**Affiliations:** 1 The Institute of Basic Medical Sciences, National Cheng Kung University, Tainan, Taiwan; 2 Leadgene Biomedical, Inc., Tainan, Taiwan; 3 Department of Medical Laboratory Science and Biotechnology, College of Medicine, National Cheng Kung University, Tainan, Taiwan; 4 National Mosquito-Borne Diseases Control Research Center, National Health Research Institutes, Tainan, Taiwan; 5 Department of Pediatrics, National Cheng Kung University Hospital, College of Medicine, National Cheng Kung University, Tainan, Taiwan; 6 Center of Infectious Disease and Signaling Research, National Cheng Kung University, Tainan, Taiwan; 7 National Institute of Infectious Diseases and Vaccinology, National Health Research Institutes, Tainan, Taiwan

**Keywords:** EV-A71, ENO1, Autoantibody, scFv

## Abstract

**Background:**

Enterovirus A71 (EV-A71) is a neurotropic virus which may cause severe neural complications, especially in infants and children. The clinical manifestations include hand-foot-and-mouth disease, herpangina, brainstem encephalitis, pulmonary edema, and other severe neurological diseases. Although there are some vaccines approved, the post-marketing surveillance is still unavailable. In addition, there is no antiviral drugs against EV-A71 available.

**Methods:**

In this study, we identified a novel antibody that could inhibit viral growth through a human single chain variable fragment (scFv) library expressed in mammalian cells and panned by infection with lethal dose of EV-A71.

**Results:**

We identified that the host protein α-enolase (ENO1) is the target of this scFv, and anti-ENO1 antibody was found to be more in mild cases than severe EV-A71 cases. Furthermore, we examined the antiviral activity in a mouse model. We found that the treatment of the identified 07-human IgG_1_ antibody increased the survival rate after virus challenge, and significantly decreased the viral RNA and the level of neural pathology in brain tissue.

**Conclusions:**

Collectively, through a promising intracellular scFv library expression and screening system, we found a potential scFv/antibody which targets host protein ENO1 and can interfere with the infection of EV-A71. The results indicate that the usage and application of this antibody may offer a potential treatment against EV-A71 infection.

## Background

Enterovirus A71 (EV-A71), a subtype virus of *Enterovirus* A species belonging to the *Enterovirus* genus in the *Picornaviridae* family, is a major causative agent of viral hand-foot-and-mouth disease (HFMD) and herpangina in infants and children. However, EV-A71 is a neurotropic virus and may develop into severe central nervous system complications, such as bulbar and brainstem encephalitis, cerebella ataxia, poliomyelitis-like paralysis, pulmonary edema, pulmonary hemorrhage, myocarditis, and cardiopulmonary collapse [1–3].

Viral loads, viral genetic determinants, host factors or sanitary conditions are all important factors which result in different clinical outcomes of EV-A71 infection. Immune response of host also plays a critical role of virus infection. The diversity of antibody specificity is enhanced by the process of somatic hypermutation and class switch recombination, antibody engineering and application of monoclonal antibodies (mAbs) become more and more powerful for therapeutic and diagnostic purposes [4]. The variable region of heavy chain (V_H_) and light chain (V_L_) consist of interlaced complementarity determining regions (CDRs) and framework region (Fw). This variable fragment (Fv) is the major and smallest binding domain to specific antigen. However, some limitations of intact antibody facilitate the application of single chain variable fragment (scFv) by joining the V_H_ and V_L_ with a flexible peptide linker. The scFv contains the whole antigen-binding activities but with the smallest steric hindrance and minimum antigenic characteristics as a candidate for further clinical drug development. There are more and more studies that utilized scFv to find the fragments against infectious agents and cancer cells. The human scFv library can be generated by non-immunized donor and select human-derived candidates directly [5, 6]. Besides, utilization of scFv against viral proteins has been extensively applied to study antiviral resolutions [7–11]. Furthermore, the methodology can also be applied to identify targets for specific mechanisms [12]. There are several platforms which display antibody or scFv libraries to screen for the candidates, and phage display is the most powerful and applicable one based on the binding characteristics between target and candidate [13, 14]. It is applied to develop antibody candidates against not only infectious agents like bacteria, viruses, and parasites, but also diagnostic or therapeutic targets or molecules [15–21]. In addition, intracellular library display system had been developed and applied to screen the candidates through cellular phenotypic activity, such as survival from virus challenge [22–24]. Therefore, in this study, we applied a phenotypic directed library display to screen and further identify antibody candidates which have the ability to inhibit EV-A71 infection.

## Methods

### Cells and virus infection

Dulbecco’s modified Eagle’s medium (Gibco) containing 10% fetal bovine serum (FBS) and 2% streptomycin-ampicillin (P/S) was used to culture RD (rhabdomyosarcoma) cells. Stock EV-A71 virus strain N4643-TW98 and CA16 was isolated from National Cheng Kung University Hospital, Tainan, Taiwan. Virus was propagated in RD cells, and their titers were determined by plaque assay and TCID_50_-ELISA in RD cells as described previously [25, 26]. To analyze expression of ENO1, cells were infected with viruses at different multiplicity of infection (M.O.I.) for 24 h and extracted mRNA for reverse transcription-PCR or Western blotting by using RIPA lysis buffer.

### Construction of human scFv library

We used a modified vector, pDisplay-STOP, from the original pDisplay vector (Invitrogen, CA). Stop codon was added into pDisplay vector before PDGFR transmembrane domain (PDGFR-TM) sequence which lead scFv to express on the surface of the cell. This modified plasmid can express both intracellular and secreted scFv in mammalian cells. Collected peripheral blood mononuclear cells (PBMCs) (Number: VR-100-131, Institutional Review Board, National Cheng Kung University Hospital, College of Medicine, National Cheng Kung University) were cultured in Lymphocyte Growth Medium-3 (LGM-3, Lonza Walkersville, Inc., MD) with proper cytokines and lactoferin cocktail (a gift from Leadgene Biomedical, Inc, Taiwan) and T cell inhibitor. The neutralizing antibody titer against EV-A71 from donors’ sera was 512. Total RNA was extracted (Genedirex, Taiwan) from B cells and reverse transcription (Leadgene, Taiwan) was performed to get cDNA for further V_H_/V_L_ amplification by polymerase chain reaction (PCR). Specific primers were modified according to the protocol from Jennifer Andris-Widhopf et al. [27]. In brief, V_H_ (~ 470 bp) and V_L_ (~ 350 bp) genes were amplified and overlapped from cDNA pool, followed by insertion into the pDisplay-STOP vector. Library size was calculated by using BstOI cleavage to evaluate the variation of scFv fragment and normalized with transformation efficiency. The original library size was approximately 2.5 × 10^8^ (data not shown).

### scFv library expression and biopanning

Empty control vector (pDisplay-STOP) and pDisplay-STOP-scFv plasmids were transfected into RD cells (HyFect™ DNA Transfection reagent, Leadgene) and selected by G418 sulfate for 1 week. These RD cells were analyzed by Western blotting or flow cytometry using mouse anti-HA tag antibody (Croyez, Taiwan) and beta actin antibody (Arigo, Taiwan) to verify generation of scFv. Before virus infection, the cells were washed several times to remove secreted scFv. The cells were then infected with EV-A71. The different multiplicity of infection (M.O.I.) were used from 0.01, 0.05, 0.1, to 0.5 at different rounds of selection. After infection, the cells were collected and washed 5 times to remove unhealthy cells. Total mRNA was extracted from the healthy cells and RT-PCR was performed. The genes of scFv or V_H_ and V_L_ were amplified and subcloned back into the pDisplay-STOP vector.

### Cell viability assay

Level of cell viability was measured by WST-8 reagent based on redox reaction (Bimake, USA) according to the manufacturer’s instructions. Briefly, 10 μL of the reagent was added per 100 μL cell medium and placed the cell culture plate back into incubator. The absorbance at 450 nm was measured after 1 h. The absorbance was normalized with non-infected cell control.

### Analysis of VH and VL gene sequence

To analyze the V_H_ and V_L_ gene, scFv sequences were cloned into pJET1.2/blunt cloning vector for further sequencing. The sequences were analyzed by Vbase2 database (http://www.dnaplot.de/vbase2) to assign homologous germline genes.

### Expression of specific scFv and intact antibody in mammalian cells

To overexpress scFv in cells, cells were transfected with scFv expressing plasmid using HyFect™ DNA transfection reagent (Leadgene, Taiwan). After 24 h, cells were lysed with RIPA buffer and analyzed by Western blot using mouse anti-HA monoclonal antibody. Intact antibody was obtained by co-transfection of two expression vectors (pcDNA3.4) containing heavy chain and light chain sequences separately in ExpiCHO system (Thermo Fisher Scientific) and purified by protein A resin.

### Co-immunoprecipitation (Co-IP) assay and mass identification analysis

To perform Co-IP assay, cell lysate was extracted from EV-A71 infected RD cells by 1% NP-40 lysis buffer. MAB979 (2 μg) (Millipore) recognizes VP0 and VP2 of EV-A71 was diluted in 50 μL of PBS and incubated with protein G magnetic beads (from 20 μL slurry solution) for 5 min followed by three washes with 1 mL of PBS. Cell lysate (500 μg in 100 μL 1% NP-40 lysis buffer) was then added into MAB979 (Millipore) captured protein G magnetic beads and incubated for 24 h at 4 °C. The beads were washed with 1 mL of PBS-T (PBS with Tween 20) for a total of 10 times, eluted with 25 μL 0.2 M Glycine buffer (pH 2.6) and neutralized with 5 μL 1 M Tris buffer (pH 9). Samples were analyzed by Western blotting using MAB979 (1:5000, Millipore) and mouse anti-HA tag antibody (1:5000, Leadgene Biomedical, Inc.). To analyze 07-IgG_1_ binding protein, 07-IgG_1_ was conjugated to NHS-activated magnetic beads (Thermo Fisher Scientific). Cell lysates (1 mg) from EV-A71 infected or non-infected RD cells were extracted by RIPA buffer and incubated with 50 μL 07-IgG_1_ magnetic beads at 4 °C for 24 h. Beads were then washed by 1 mL PBS-T 10 times. Bound proteins were eluted by using 25 μL of 0.2 M Glycine buffer (pH 2.6) and neutralized with 5 μL of 1 M Tris buffer (pH 9). Samples were analyzed by 4–15% SDS-PAGE (SMObio, Taiwan). For protein identification, gel fraction was sent for LC–MS/MS analysis by OmicsLab (Taiwan). The result was analyzed using Proteome Discoverer software (version 1.4, thermo Fisher Scientific).

### Enzyme-linked immunosorbent assay (ELISA)

Proteins (5 μg/mL) were diluted in 0.05 M Carbonate-Bicarbonate (pH 9.6) and coated on 96-well ELISA plates (GeneDireX) at 4 °C for 16 h. Plates were blocked with 1% BSA in PBS for 1 h followed by washing with PBST (PBS with 0.05% Tween 20) for three times. Antibodies or sera (1:100 dilution) were diluted, added to wells at 37 °C for 1 h and washed with PBST. For ENO1 antibody detection, 07-IgG_1_ purified as described above was used as control. Bound antibodies were detected by horseradish peroxidase (HRP)-conjugated goat anti-human IgG antibody (Croyez, Taiwan). After final washes, color development was performed by the addition of 50 μl TMB substrate, and the reaction was stopped by the addition of an equal volume of 2 N sulfuric acid. The optical density (OD) at 450 nm was measured.

### Viral growth inhibition assay in conjunction with ELISA detection

The principle of EV-A71 detection method was adapted from microneutralization-enzyme-linked immunosorbent assay (microNT ELISA) described previously [25, 28]. RD cells were seeded at 10^4^/well 24 h before transfection. Transfection of pTT5-scFv07 and pTT5 vector only was performed according to the manufacturer’s instruction of HyFect™ Transfection Reagent (Leadgene, Taiwan). After transfection for 48 h, 1TCID_50_ or 10TCID_50_ of EV-A71 (N4643-TW98 strain) was added to the transfected cells. At 24 h post-infection, ELISA was performed to detect viral antigen. Cells were fixed with 80% cold acetone and then washed with PBS-T. Followed by incubation with mouse anti-EV-A71 monoclonal antibody (MAB979, 1:2000; Millipore) at room temperature for 1 h, cells were incubated with secondary antibody: peroxidase-labeled antibody against mouse IgG (pre-adsorbed, 1:4,000; Genetex), at room temperature for 1 h. Wells were washed again with PBS-T and subsequently added 3,3′,5,5′-tetramethylbenzidine substrate for 5 min, then stopped by 1 N sulfuric acid solution. Finally, the absorbance was measured at 450 nm (A450) and normalized with non-infected cell control.

### EV-A71 infected ICR suckling mouse model

Breeder mice of the ICR strain were purchased from BioLasco Taiwan Co. Ltd, and 6-day-old ICR suckling mice were randomly divided into five groups: group 1, intracerebrally injected with 5 × 10^4^ pfu/mouse of 60 °C heat-inactivated EV-A71 (iEV-A71, n = 3); group 2, intracerebrally injected with 5 × 10^4^ pfu/mouse of live EV-A71 and saline (EV-A71, n = 3); group 3, intracerebrally injected with 5 × 10^4^ pfu/mouse of live EV-A71 and 10 mg/kg of isotype control antibody (BioXcell, West Lebanon, NH) (Isotype control, n = 6); group 4, intracerebrally injected with 5 × 10^4^ pfu/mouse of live EV-A71 and 10 mg/kg of clone 07 recombinant antibody (07-IgG_1_, n = 6); and group 5, intracerebrally injected with 5 × 10^4^ pfu/mouse of live EV-A71 and 2 mg/kg of recombinant human ENO1 protein (Leadgene, Taiwan) (rhENO1, n = 6). All experiments were followed regulation of IACUC (No. 108064) (SIDSCO Inc., Taiwan). The clinical scores were recorded according to the illness symptoms as follows: 0, healthy; 1, reduced mobility; 2, wasting; 3, limb weakness; 4, limb paralysis; and 5, death.

### Detection of RNA expression level by PCR or quantitative real-time PCR

Total cellular RNA of brain tissue of each suckling mice was extracted using Ambion TRIzol reagent (Thermo Fisher Scientific, Waltham, MA, USA) according to the manufacturer’s instructions. After being transcribed to cDNA by M-MLV reverse transcriptase (Promega, Madison, WI, USA) with specific viral and oligo dT primers, the levels of ENO1 and EV-A71 RNA were determined using the ABI Step One Real-Time PCR system (Applied Biosystems, Foster City, CA, USA) or regular PCR (ABI 9700) with specific primers. The comparative threshold cycle (Ct) value was normalized to the endogenous glyceradehyde-3-phosphate dehydrogenase (GAPDH) mRNA level. Primer sequences used for PCR analysis were as below: 5′ human ENO1 (5′-GTACCGCCACATCGCTGACTTG-3′); 3′ human ENO1 (5′-AGCATGAGAACC GCCATTGATGAC-3′); 5′ human beta actin (5′-AGCACAGAGCCTCGCCTT-3′); 3′ human beta actin (5′-CATCATCCATGGTGAGCTGG-3′); 5′ EV-A71 (5′-AATCTCTC GCATGGCAAACT-3′); 3′ EV-A71 (5′-CAGTCCGCACTGAGAACGTA-3′); 5′ mouse GAPDH (5′- CCATGCCATCACTGCCACCC-3′); 3′ mouse GAPDH (5′-GCCATGCC AGTGAGCTTCCC-3'); EV-A71 -RT (5′-ATAGCTCCGGACTGCTGTCC-3′).

### Histopathology examination of animal tissue

The brain tissues were collected from suckling mice and fixed with 10% formaldehyde for 48 h. The tissues were embedded in paraffin and cut into 3 μm-thick sections on slides for Hematoxylin/Eosin (H&E) and immunohistochemical (IHC) staining. The tissues were incubated with rabbit anti-ENO1 (1:100; GeneTex, CA, USA) or rabbit anti-EV-A71 (1:500; GeneTex, CA, USA) antibodies at 37 °C for 1 h and then with Goat anti-Rabbit IgG (H + L) as a secondary antibody (1:50; Leadgene Biomedical, Tainan, Taiwan) at room temperature for 30 min. The stained slides were photographed using a light microscope equipped with a Motic EasyScan Pro (NA 0.75, Canada) for morphometric analysis.

## Results

### Establishment of human scFv library and screening for anti-EV-A71 scFv

**Fig. 1:**
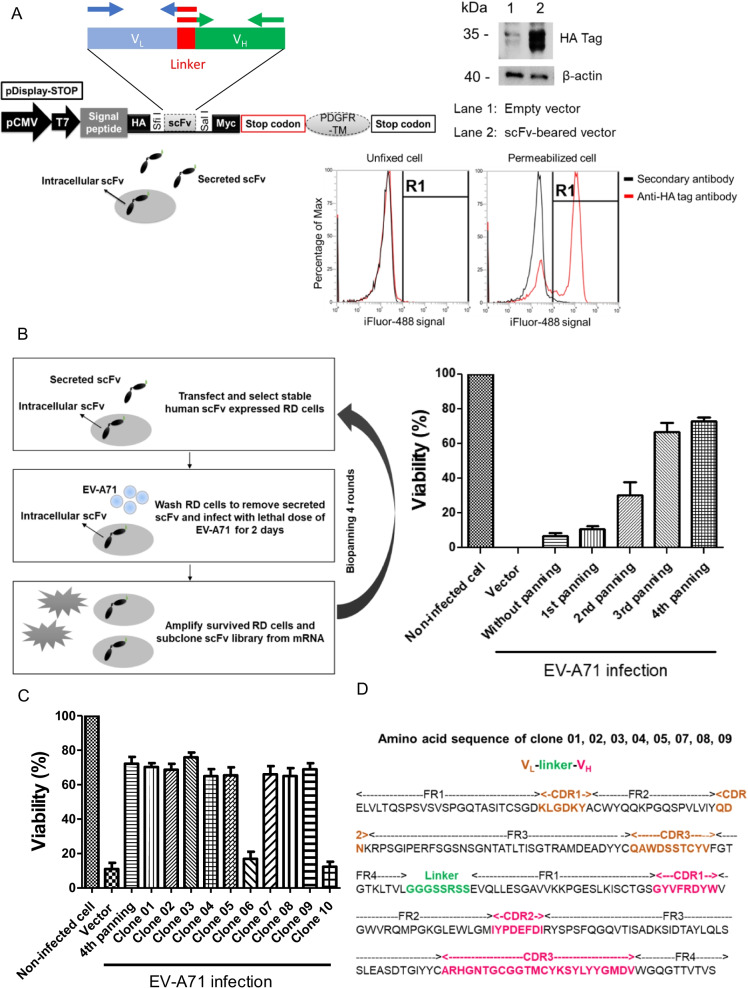
Construction of human scFv library into pDisplay-STOP vector, expression of scFv in RD cells, and panning for candidates against EV-A71 infection. **A** Modified pDisplay-STOP vector with stop codon before PDGFR transmembrane domain sequence in the original pDisplay vector. Expression of scFv in cells was validated by using HA tag antibody on Western blotting and flow cytometric assay **B** Left figure: flowchart of biopanning. Right figure: Total RNA was extracted and reverse transcribed from surviving cells of different panning rounds, and amplified scFv genes were cloned into pDisplay-STOP vector. RD cells were transfected with the libraries from different rounds and challenged with EV-A71 (M.O.I. = 0.01). After 48 h of infection, WST-8 reduction assay was performed to measure the cell viability. **C** Cell viability after EV-A71 infection of cells expressed with 10 scFv clones derived from 4th biopanning. Total RNA was extracted and reverse transcribed from surviving cells after 4th panning round, and amplified scFv genes were cloned into the pDisplay-STOP vector. RD cells were transfected with 10 of the different clones from the 4th panning rounds and challenged with EV-A71 (M.O.I. = 0.01). Cell viability was examined after 36 h of infection. **D** Amino acid sequences of anti-EV-A71 scFv. Clones 01, 02, 03, 04, 05, 07, 08, and 09 derived from the 4th biopanning possessed the same amino acid sequence

For generation of intracellular scFv library, we modified a vector name pDisplay-STOP from the original pDisplay vector. This original system is suitable for specific antigen selection to sort scFv-expressed cells. However, it may not be easy to identify whether these scFvs could protect against EV-A71 infection in cells. The stop codon was added before PDGFR-TM sequence to obtain the modified pDisplay-STOP vector (Fig. 1A). This modified plasmid can express both intracellular and secreted scFv in mammalian cells. After the removal of supernatants, the scFv which exist in intracellular compartment can help us to screen for antibody against EV-A71 after infection.

We collected blood sample from a healthy adult who had high neutralizing antibody titer (512x) against EV-A71. Total RNA extraction from buffy coat and reverse transcription were performed to get cDNA. V_H_ (~ 470 bp) and V_L_ (~ 350 bp) genes were amplified from the cDNA pool. The purified V_H_ and V_L_ DNA were overlapped by PCR and inserted into the pDisplay-STOP vector. Empty control vector (pDisplay-STOP) and pDisplay-STOP-scFv plasmids were transfected into RD cells and selected by G418 sulfate for 1 week. We found that these RD cells did express the scFv by using anti-HA tag antibody on Western blotting and flow cytometric assay (Fig. 1A). These scFv were detected in permeabilized cells but not on the surface of unfixed cells. These data demonstrated that we have generated scFv library and expressed in cells successfully.

The flowchart of biopanning was shown in Fig. 1B (left figure). To expand the library of scFv, we amplified scFv DNA from survived cells after EV-A71 infection. V_H_ and V_L_ DNA were amplified separately, and scFv gene repertories were further created by random combination of these V_H_ and V_L_ using overlapping PCR. This procedure might increase the possibility to screen highly potent scFv against EV-A71 infection. In the first round of selection, we used M.O.I. at 0.01 for infection. After 48 h, all the control cells were dead. There were around 5–10% of scFv-expressed cells which survived (right figure, Fig. 1B). After four rounds of panning procedures, survival rate of cells reached 70–80% (Fig. 1C).

### Intracellular scFv07 decreased EV-A71 viral growth

After the biopanning, we amplified the scFv genes that existed in surviving cells and identified 10 single clones. These clones were individually transfected to RD cells and characterized the cell viability after infection with EV-A71. We found that clones 1, 2, 3, 4, 5, 7, 8, and 9 had similar viability with the scFv pool from the 4th panning round (Fig. 1C). Therefore, we analyzed the sequences of these clones through sequencing and online software. We found that the gene sequences of V_H_ and V_L_ were all mapped to the same gene among these 8 clones. The amino acid sequences of the frames and CDRs of V_H_ and V_L_ were shown in Fig. 1D. Since we identified a single candidate gene clone (we chose clone07) which may have the potential to inhibit viral growth, we further proceeded with codon optimization and synthesized the sequence with longer linkers (from GGGSRS to GGGGSGGGGSGGGGS) in order to sustain the function of scFv, and cloned into another vector (pTT5) which have better yields for protein expression in mammalian cells. We also confirmed the expression of scFv by using anti-HA antibody to detect the HA tag at C-terminus (data not shown).

**Fig. 2:**
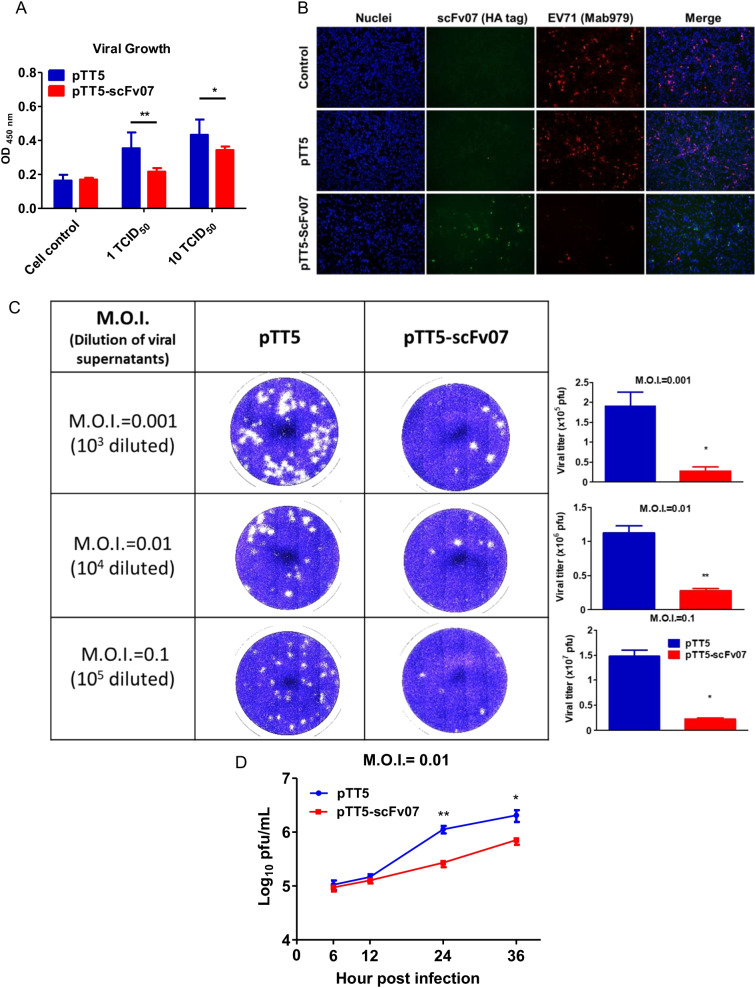
Inhibition of viral growth by scFv07 in cells. **A** RD cells were infected with different titers (0, 1, and 10 TCID_50_) of EV-A71 at 2 days post-transfection with scFv07 expressing plasmid (pTT5-scFv07) or pTT5 vector control. After 24 h of infection, the cells were fixed, and ELISA was performed by using anti-EV-A71 antibody to determine the expression of viral antigen. The absorbance of 450 nm was determined. **B** Post-transfection with scFv07 expressing pTT5-scFv07 plasmid or pTT5 vector cells were infected with EV-A71 (M.O.I. = 0.1) for 24 h followed by staining with anti-HA tag and EV-A71 antibody (MAB979). Magnification: 10x. **C** Viral supernatants from above EV-A71-infected cells as indicated were used to calculate virus titer by plaque assay. **D **Viral growth (M.O.I. = 0.01) of pTT5 or pTT5-scFv07 expressed RD cell were examined at 6, 12, 24 and 36 h post infection by plaque assay. *p < 0.05; **p < 0.001

We examined the interference of viral growth by intracellular scFv07 on ELISA platform which detect the viral antigen after infection. We found significantly less viral antigens were detected in the cells with scFv expression than the cells only transfected with pTT5 vector control (Fig. 2A). IFA data also indicated that overexpressed scFv07 in cell reduced EV-A71 replication (Fig. 2B). In addition, we further examined viral titers from supernatant of EV-A71-infected pTT5- or pTT5-scFv07-transfected cells, and a much lower viral titer was seen in pTT5-scFv07-transfected cells (Fig. 2C). The virus growth curve was determined in scFv07-transfected cells and showed significantly slower viral growth post-infection when compared with vector-transfected cells (Fig. 2D). These results demonstrated that scFv07 has the ability to inhibit EV-A71 viral growth, and the mechanism of anti-virus was characterized further.

### scFv07 and intact 07-human IgG_1_ do not recognize EV-A71 proteins

**Fig. 3:**
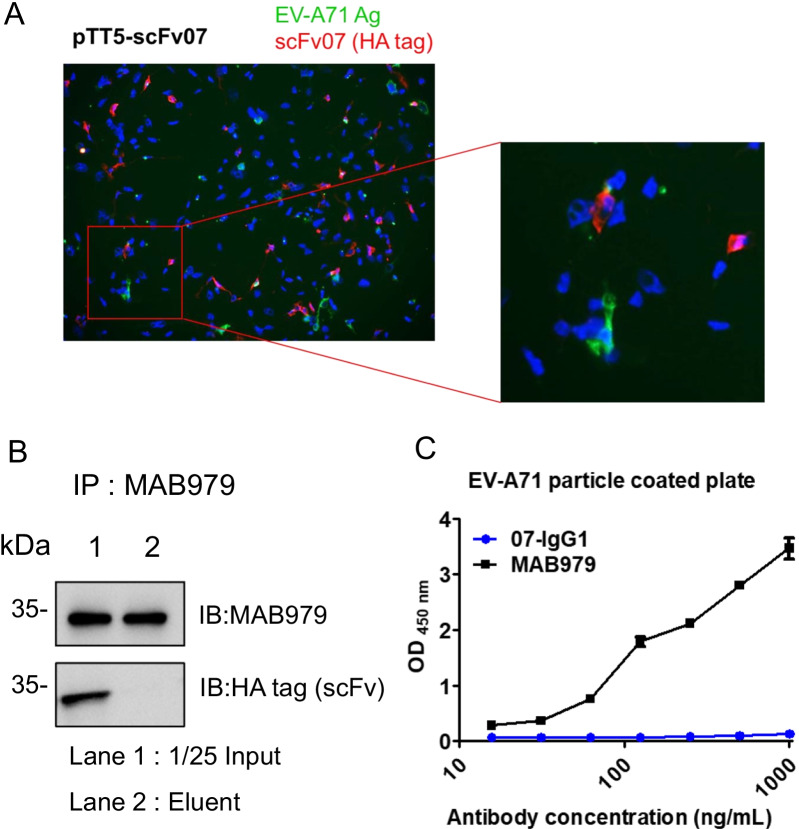
Intracellular scFv07 and 07-IgG_1_ do not recognize EV-A71 capsid proteins. **A** Cells were infected with EV-A71 (MOI 0.1) for 24 h followed by staining with rabbit anti-HA tag and mouse anti-EV-A71 (Mab979) antibody. Goat anti-mouse IgG-488 and goat anti-rabbit IgG-594 were used for further detection. **B** EV-A71 infected cell lysate (500 μg) was used for immunoprecipitation assays. MAB979 (2 μg) was captured by protein G magnetic beads (20 μL) and incubated with lysate for 24 h at 4 °C. After several washes, eluents were analyzed by WB using MAB979 and anti-HA tag antibody. **C** PEG precipitated EV-A71 were coated onto ELISA wells. Serial dilution of 07-IgG_1_ and MAB979 were added as primary antibody and incubated at 37 °C for 1 h. HRP labeled goat anti-mouse and human IgG were used for detecting bound antibody

To explore the relationship between scFv07 and EV-A71, scFv07 expressed RD cells were infected with virus and analyzed its expression using IFA. As shown in Fig. 3A, we found EV-A71 which was stained by MAB979, an antibody for VP0 and VP2, was not colocalized with scFv07. Moreover, by using co-immunoprecipitation assays, we found that MAB979 trapped VP protein did not interact with scFv07 in RD cells (Fig. 3B). To validate whether intact IgG shows any difference with scFv, we expressed and purified 07-IgG_1_ for further experiments. We used PEG-precipitated EV-A71 for indirect ELISA. The result indicated that MAB979, instead of 07-IgG_1_, could bind to EV-A71 protein in a dose-dependent manner (Fig. 3C). The result suggests that scFv07 or 07-IgG_1_ does not recognize EV-A71 capsid proteins.

### Characterization of 07-IgG_1_ binding protein in cells

**Fig. 4:**
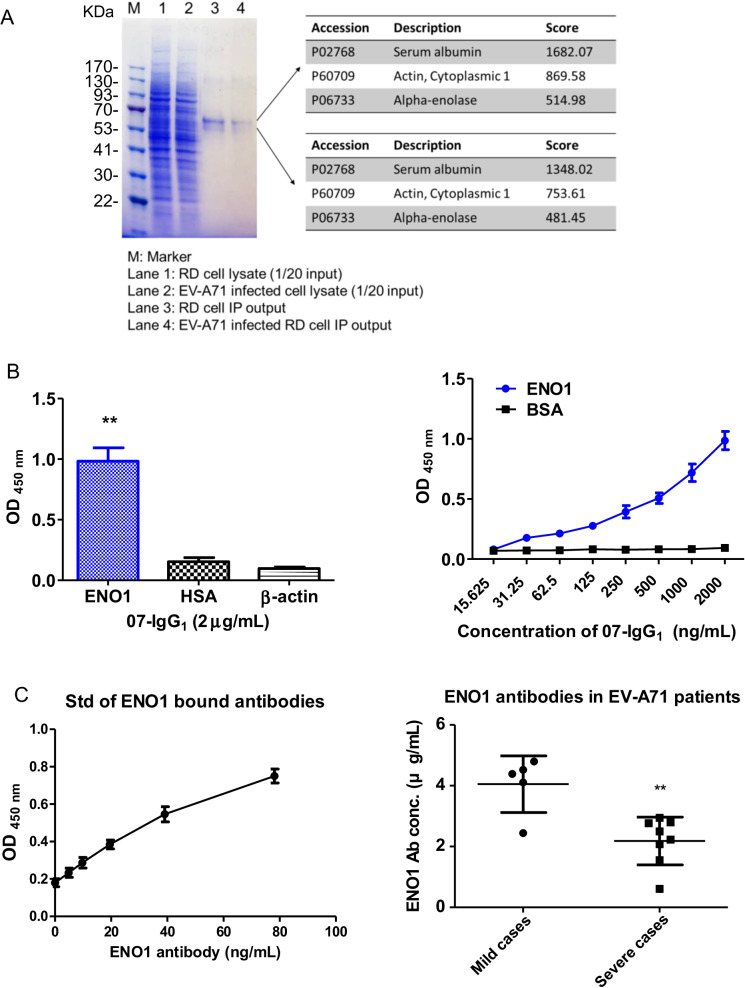
Characterization of 07-IgG_1_ using mass spectrum identification. **A** Mass spectrum identification of 07-IgG_1_ pull-down proteins could be HSA, β-actin, or ENO1. **B** ENO1, HSA, or β-actin recombinant protein was used in indirect ELISA with 07-IgG_1_. **C** EV-A71 patients’ sera were used to detect IgG against hENO1 protein. The levels of anti-ENO1 antibody were quantified by ELISA using 07-IgG_1_ as the standard antibody. **p < 0.001

To further identify the target protein of 07-IgG_1_ antibody, we conjugated 07-IgG_1_ to sepharose6B through covalent bound manner to perform pull-down assay. In Fig. 4A, we found similar pull-down pattern in RD cell lysate (Lane 3) and EV-A71-infected RD cell lysate (Lane 4) on SDS-PAGE. Extracted gels were further analyzed by mass spectrometry (MS), and human serum albumin, β-actin (Actin, Cytoplasmic 1), and ENO1 (Alpha-enolase) were found to interact with 07-IgG_1_. These proteins may either be a complex or a nonspecific binding protein. We further used individual protein to check the binding ability with 07-IgG_1_ by using indirect ELISA. We found only ENO1 can significantly bind to 07-IgG_1_ (Fig. 4B). The dose-dependent binding curve proved a specific binding response. Thus, 07-IgG_1_ was an anti-ENO1 antibody. To examine whether patient serum contain anti-ENO1 antibody, we found that mild infection cases contained higher anti-ENO1 titer than severe cases (Fig. 4C). These results imply 07-IgG_1_ identified from B cells might correlate to the severity of EV-A71 infection.

### Investigation of the role of ENO1 during EV-A71 infection in cells

**Fig. 5:**
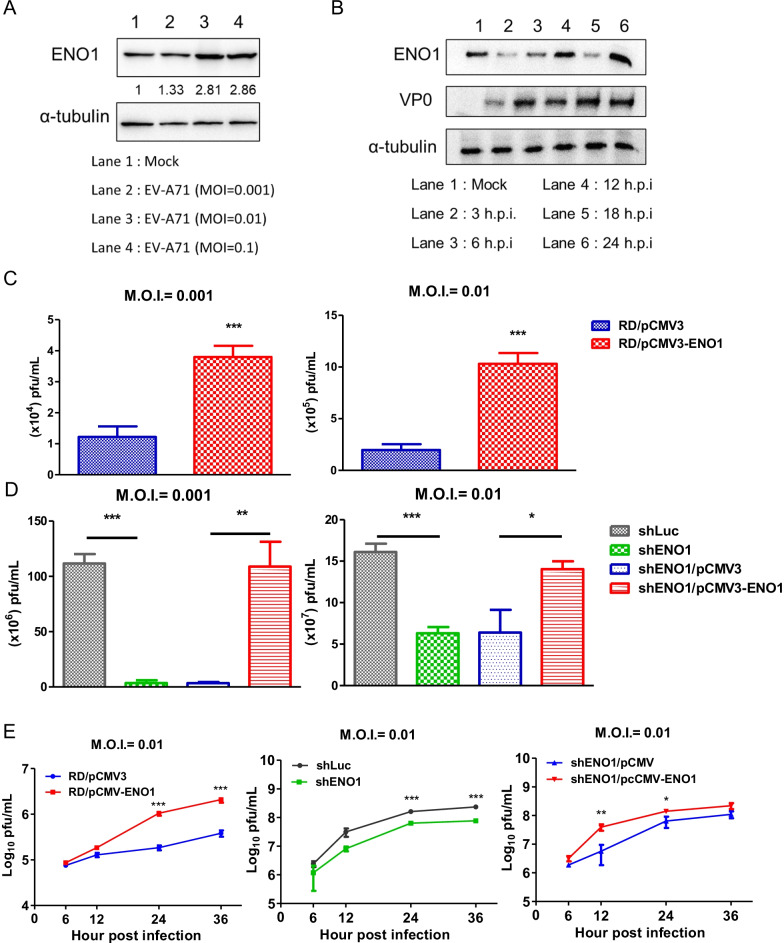
ENO1 is upregulated in EV-A71 infection and enhances viral replication. **A** ENO1 protein expression of EV-A71 infected RD cells were examined by WB. The fold change of ENO1 was normalized with the intensity of α-tubulin. **B** RD cells were infected EV-A71 (M.O.I. = 0.01) and ENO1 expression was analyzed at different hours post infection (h.p.i.). **C** ENO1 overexpressed RD cells were infected with EV-A71 for 24 h at the indicated M.O.I. of 0.001 and 0.01. Virus titers in the supernatant were determined by plaque assay. **D** ENO1 knock-down or supplement with ENO1 RD cells were infected with EV-A71, and viral titers were determined by plaque assay. **E** ENO1 overexpressed or knock-down RD cells were infected with EV-A71 at M.O.I. of 0.01 and virus growth was analyzed by plaque assay. *p < 0.05; **p < 0.001, ***p < 0.0001

To identify the role of ENO1 in EV-A71 infection, RD cells were infected with EV-A71 for 24 h from 0.001 to 0.01 M.O.I.. The fold-change of ENO1 protein was normalized with self α-tubulin by Western blotting. The expression of ENO1 was found to be increased in a dose-dependent manner (Fig. 5A). We also infected RD cells at 0.01 M.O.I. and analyzed ENO1 protein expression at different h.p.i. ENO1 protein expression was changed kinetically through the time-course (Fig. 5B). The viral titer at different M.O.I. of EV-A71 infection were all found to be significantly increased in ENO1 overexpressed cells (Fig. 5C). Furthermore, knock-down ENO1 cells showed reduced viral replication but can be rescued by adding ENO1 (Fig. 5D). Furthermore, viral growth curves (at M.O.I. of 0.01) of ENO1 overexpressed or knock-down RD cells were examined and found consistent results (Fig. 5E).

### 07-IgG_1_ protected against EV-A71 infection in ICR sucking mice

**Fig. 6:**
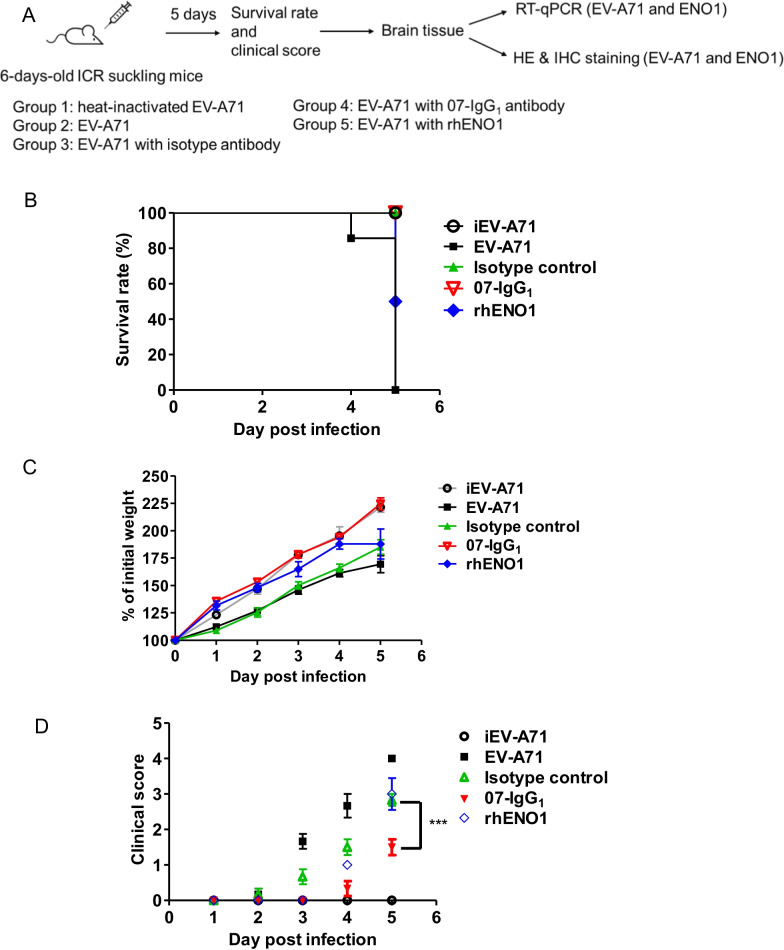
Effects of 07-IgG_1_ upon EV-A71 infection in animal model. **A** Scheme of the experimental design using 6-days-old ICR sucking mice. **B** Survival rate, **C** Percentage of initial body weight, and **D** clinical score of each suckling mice was recorded for 5 days. ***p < 0.0001

**Fig. 7:**
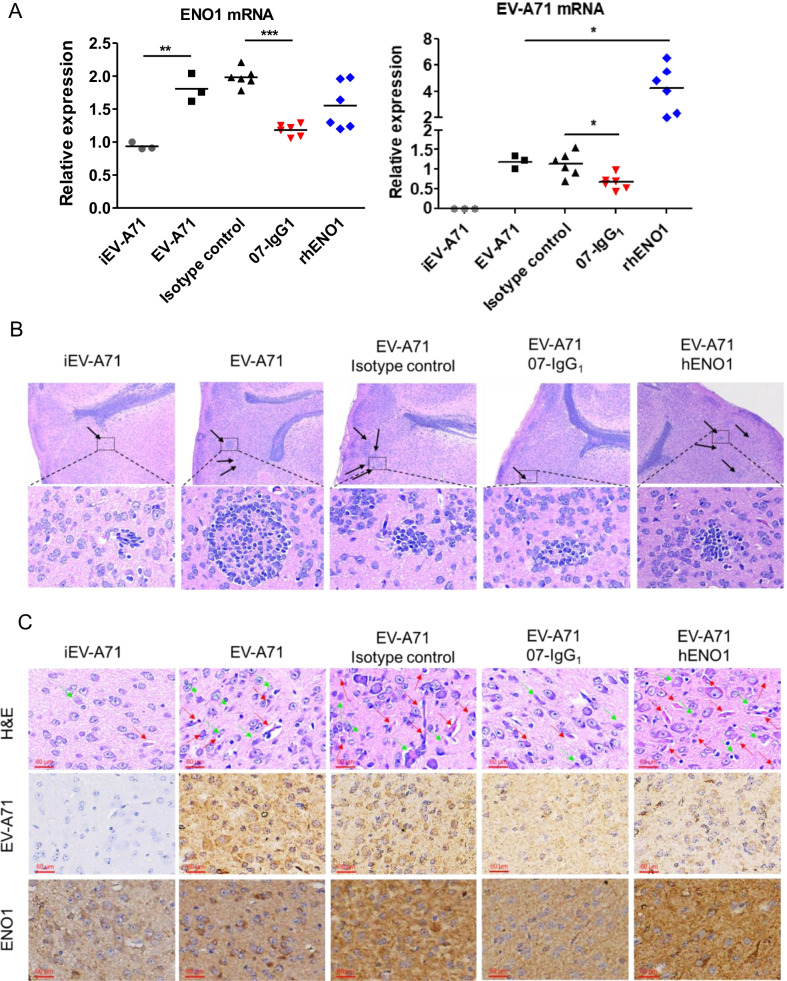
07-IgG_1_ inhibits EV-A71 replication in mice. **A** Brain tissues from sacrificed mice were used for ENO1 and EV-A71 qPCR. **B** H&E stain was performed to analyze ventral striatum immune cells infiltration. Upper panel: 4 × objective; Lower panel: 40 × objective. Black arrows indicate the cluster of immune cell infiltration. **C** H&E and IHC stain of brainstem from sacrificed mice. Red arrows indicate gliosis and green arrows indicate necrotic and partially lytic forms of basophilic neuronal necrosis. *p < 0.05; **p < 0.001, ***p < 0.0001

To evaluate whether 07-IgG_1_ can protect mice from EV-A71 infection in vivo, 6-days-old ICR suckling mice were used for EV-A71 MP4 strain challenge. As shown in Fig. 6A, we randomly divided the mice into 5 groups. All mice were intracerebrally (i.c.) injected with EV-A71 MP4 strain (5 × 10^4^ pfu), with or without isotype control or 07-IgG_1_ (10 mg/kg), and recombinant hENO1 (2 mg/kg). As shown in Fig. 6B, mice showed 100% survival rate in inactivated EV-A71 (iEV-A71), isotype control, and 07-IgG_1_ group. Mice with recombinant hENO1 showed a 50% reduction of survival rate at day 5. Although isotype control shows a 100% survival rate at day 5, the body weight decreased and a clinical score increase were seen after EV-A71 infection (Fig. 6C and D). In the mice group treated with 07-IgG_1_, the body weight of mice were higher and clinical scores were lower compared with other groups (Fig. 6C and D). The EV-A71 and ENO1 RNA expression levels of the mouse brain were measured by using RT-qPCR (Fig. 7A). Mice injected with heat-inactivated EV-A71 (iEV-A71) were used as mock infection control. The relative expression of EV-A71 RNA was found to decrease significantly in the 07-IgG_1_ group as compared with isotype control group. EV-A71 infection with rhENO1 treatment showed a dramatical increase of viral mRNA in mice brain tissue. Notably, EV-A71 infection up-regulated ENO1 mRNA expression which could be reduced upon 07-IgG_1_ treatment. However, mice infected with EV-A71 infection in the presence of rhENO1 showed no statistically significant difference of ENO1 mRNA expression. To evaluate the histopathology, the brain tissues were collected and embedded in paraffin for further Hematoxylin and Eosin (H&E) and immunohistochemical (IHC) staining. As shown in Fig. 7B, EV-A71-infected mice showed increased clusters of inflammatory cells infiltration. 07-IgG_1_-treated mice showed less clusters than isotype control group. In brainstem, EV-A71-infected mice were found to have increased gliosis and necrotic forms of basophilic neuronal necrosis (Upper panel, Fig. 7C). In contrast, the phenomena were reduced in the presence of 07-IgG_1_. The expression of EV-A71 and ENO1 in the brainstem was examined by IHC (Middle and lower panel, Fig. 7C). Mice treated with 07-IgG_1_ showed less EV-A71 and ENO1 expression which is similar with the result of RT-qPCR in Fig. 7A. Taken together, our results suggest that ENO1 play a crucial role in EV-A71 infection. Inhibition of ENO1 by 07-IgG_1_ can reduce EV-A71 induced pathology and viral replication.

## Discussion

In the present study, we constructed scFv library to screen functional antibody against EV-A71 replication. Although sera from donors showed neutralizing titers against EV-A71, it is unclear whether these antibodies recognize with EV-A71 proteins or belong to polyreactive antibodies [29]. We display scFv library in mammalian cell to establish phenotypic directed screening platform rather than traditional library display system based on binding characteristics. It means antibodies biopanning from our library platform might not recognize EV-A71 but might have the ability to interfere with viral replication. Based on the advantages of intracellular library, Xie et al. [22] used a lentivirus system to introduce an antibody library into HeLa cells and challenged with human rhinovirus to select the antibody candidate which can inhibit viral infection intracellularly.

The candidate antibody, scFv07 or 07-IgG_1_, was identified by its inhibition in viral replication. The virus replication was reduced under scFv07 expression in cells or adding 07-IgG_1_ as an exogenous treatment. This implies Fc of this candidate antibody is not necessary as functional bioactivities against the virus. To our surprise, using mass spectrometry identification, we found that 07-IgG_1_ actually recognizes host protein ENO1 instead of EV-A71 protein. There have been several studies which showed that host factors were critically involved during viral infection [30, 31]. Kuo et al. [32] revealed that host protein- E3 ubiquitin − protein ligase NEDD4-like (NEDD4L) is upregulated upon EV-A71 infection. Accordingly, they also demonstrated that depletion of NEDD4L significantly reduced the replication of EV-A71 via the increase of IFN-β transcription.

ENO1 is a multifunctional protein involved in several physiological functions [33]. ENO1 has been shown to regulate human immunodeficiency virus type 1 (HIV-1) replication through glycolysis [34, 35]. Under ENO1 overexpression, HIV-1 replication is repressed. The regulations of ENO1 in different viruses are quite different. There are several reports which showed that ENO1 may play influential roles during viral infection by protein interactome analysis in EV-A71 infection [36–38]. However, there is no report of the effect of ENO1 or anti-ENO1 antibody in EV-A71 infection. We have examined that 07-IgG_1_ has no direct effect on ENO1 enzyme activity (data not shown). In hepatitis B virus infection, knock-down ENO1 expression can reduce viral replication by up-regulating type I interferon [39]. We used 07-IgG_1_ to co-treat with EV-A71 infected RD cell and found no significant change of IFN-α secretion. In addition, no phosphorylation of stat1 level change was found in EV-A71 infected shLuc and shENO1 cells (data not shown). In addition to its traditional glycolytic function, it is also found on the surface of some hematopoietic cells and epithelial cells with fibrinolytic activity [40, 41]. ENO1 is also an endothelial cell hypoxic stress protein, which is up-regulated upon hypoxia tolerance [42, 43]. Furthermore, it was found that upregulation of ENO1 was correlated with the progression of neuroblastoma, and hepatocellular carcinoma [44–47]. However, ENO1 was also reported as an autoantigen. Anti-α-enolase antibodies can be frequently detected in systemic autoimmune disorders [48–51]. Furthermore, some studies also showed that the level of ENO1 antibody was as a diagnostic or disease progression marker in lung cancer and breast cancer [52–54]. Therefore, ENO1 plays an important role in several biological, physiological, and pathologic processes [33]. In this study, we found that anti-ENO1 antibody played a role against EV-A71 infection. Han et al. [55] identified the host factor ATP6V0C which served as a target for anti-EV-A71 drug candidate. ATP6V0C (Vacuolar ATPase c subunit) is a transmembrane protein that translocates protons between plasma membranes and plays a crucial role in extracellular microenvironment formation which is involved in invasion and metastasis of cancer [56, 57]. Indeed, these results imply inhibition of cellular functionality targets which might result in reduced EV-A71 infection and replication.

Besides, ENO1 autoantibodies were found to exist in several diseases including autoimmune diseases and viral infections [48–50, 58, 59]. ENO1 was also found to be increased in cells infected with EV-A71 [36]. However, it is unclear the role of ENO1 in EV-A71. We found both mRNA and protein levels of ENO1 were upregulated in EV-A71 but not CA16 infection. Overexpression of ENO1 enhanced EV-A71 infection. In contrast, ENO1 knock-down, scFv07 overexpression, or 07-IgG_1_ treatment could inhibit EV-A71 replication in vitro and in vivo. However, there are also studies which in contrast showed that the expression of ENO1 was decreased during EV-A71 infection, and ENO1-knocked down resulted in increased viral titers in culture supernatant [38]. The difference in results may presumably be due to the ENO1 protein being involved in various important metabolic systems, and the varying time points used under different experimental conditions and systems.

In EV-A71 infected ICR suckling mice, 6-day-old mice were used for experiments and found that all infected mice were dead at day 5. To compare the levels of ENO1 and EV-A71 mRNA, we sacrificed mice and found both mRNA levels were lower in 07-IgG_1_ injected EV-A71 infected mice. Interestingly, we found that mice infected with isotype control antibody can also survive from lethal challenge at day 5. The isotype control antibodies were purified from human myeloma cell which may have some anti-inflammatory activity similar to the effect of intravenous immunoglobulins (IVIGs) in human [60]. In addition, previous study has found IVIGs could affect EV-A71 infection in mice [61]. It might be a reason as to why isotype control mice showed 100% survival rates at day 5 post-infection. In in vitro assays, we found that ENO1 overexpression showed higher EV-A71 replication, and the viral replication was down-regulated in ENO1 knockdown RD cells. In in vivo assay, we used rhENO1 to treat mice and found that viral mRNA was dramatically increased in the brain. Results are consistent in both cell and animal studies. rhENO1 administration resulted in an increased clinical score after day 4 and the survival rate drops to 50% at day 5 post-infection. The mechanism of rhENO1 to increase the survival rate is unclear. In mice, multiple factors including different functional systemic networks and immune response might be involved in EV-A71 infection as we discussed above. In the future, the effect of ENO1 during EV-A71 infection in mice will be dissected.

Our results showed that mild infection had higher antibody titer against with ENO1, in contrast to severe cases with low titer against ENO1. However, a series of studies and a cohort study are still needed to evaluate whether anti-ENO1 antibody titer can be a new indicator for the progress of EV-A71 infection. In this study, since there no commutable reference materials available, we used 07-IgG_1_ as an antibody standard for detecting ENO1 reactive human antibodies in sera. Polyclonal antibodies from ENO1 immunized animal sera belong to high affinity and host difference which might not be a suitable commutable control. Ideally, it should use native purified autoantibodies against ENO1 as standard to reflect the situation and titer in patients. Interestingly, autophagy may bridge the mechanisms between host ENO1 and viral replication since there are some studies showed that viral infection promotes autophagy, which enhances viral replication [62–64]. We also found that the expression of markers, Beclin 1 and LC3B, involved in autophagy activity were appropriately decreased with the treatment of 07-IgG_1_ in the EV-A71 mice infection model (data not shown). Moreover, ENO1 was identified to promote the self-renewal and malignant phenotype of lung cancer stem cells by AMPK/mTOR pathway [65], and MPK/mTOR pathway is also the upstream initiator during the onset of autophagy mechanism [66]. Additionally, in cellular migration and inflammation, ENO1 also play**s** a role in cell-to-cell transmission via exosomes [67], and there are more and more studies found the crosstalk between exosome and autophagy in maintenance of cellular homeostasis [68–70]. However, more studies and evidence are still needed to dissect the mechanisms among the factors.

## Conclusions

Our study applied a phenotypic intracellular library display system to identify anti-EV-A71 agent, and we have explored the role of ENO1 and anti-ENO1 antibody in EV-A71 infection. Since ENO1 antibodies have been proposed as a drug in cancers and autoimmune diseases [71], in regard to EV-A71 antiviral therapy, ENO1 targeting might be a therapeutic strategy for preventing severe disease of EV-A71 infection.

## Data Availability

The datasets used and/or analyzed during the current study are available from the corresponding author on reasonable request.

## Abbreviations

EV-A71: Enterovirus A71
scFv: Single chain variable fragment
ENO1: α-Enolase
HFMD: Hand-foot-and-mouth disease
mAbs: Monoclonal antibodies (mAbs)
V_H_: Variable region of heavy chain
V_L_: Variable region of light chain
CDRs: Complementarity determining regions
Fw: Framework region
Fv: Variable fragment
FBS: Fetal bovine serum
P/S: Streptomycin-ampicillin
RD cells: Rhabdomyosarcoma cells
PBMCs: Peripheral blood mononuclear cells
M.O.I.: Multiplicity of infection
microNT ELISA: Microneutralization-enzyme-linked immunosorbent assay
GAPDH: Glyceradehyde-3-phosphate dehydrogenase

## References

[1] Wang SM, Liu CC (2014). Update of enterovirus 71 infection: epidemiology, pathogenesis and vaccine. Expert Rev Anti Infect Ther.

[2] McMinn PC (2014). Enterovirus vaccines for an emerging cause of brain-stem encephalitis. N Engl J Med.

[3] Ho M, Chen ER, Hsu KH, Twu SJ, Chen KT, Tsai SF (1999). An epidemic of enterovirus 71 infection in Taiwan. Taiwan Enterovirus Epidemic Working Group. N Engl J Med.

[4] Rogozin IB, Solovyov VV, Kolchanov NA (1991). Somatic hypermutagenesis in immunoglobulin genes. I. Correlation between somatic mutations and repeats. Somatic mutation properties and clonal selection. Biochem Biophys Acta.

[5] Liu Y, Chang J, Chen Y, Wan B, Wang Y, Zhang G (2012). Construction of a human scFv antibody library with VH regions randomized and its application. Biotech Lett.

[6] Kulkeaw K, Sakolvaree Y, Srimanote P, Tongtawe P, Maneewatch S, Sookrung N (2009). Human monoclonal ScFv neutralize lethal Thai cobra, Naja kaouthia, neurotoxin. J Proteomics.

[7] Mukhtar MM, Li S, Li W, Wan T, Mu Y, Wei W (2009). Single-chain intracellular antibodies inhibit influenza virus replication by disrupting interaction of proteins involved in viral replication and transcription. Int J Biochem Cell Biol.

[8] Duan L, Laughlin MA, Oakes JW, Pomerantz RJ (1998). Potent inhibition of human immunodeficiency virus type 1 replication by an intracellular anti-Rev single-chain antibody. Proc Natl Acad Sci USA.

[9] Marasco WA, LaVecchio J, Winkler A (1999). Human anti-HIV-1 tat sFv intrabodies for gene therapy of advanced HIV-1-infection and AIDS. J Immunol Methods.

[10] de Carvalho NC, Williamson RA, Parren PW, Lundkvist A, Burton DR, Bjorling E (2002). Neutralizing human Fab fragments against measles virus recovered by phage display. J Virol.

[11] Vascotto F, Campagna M, Visintin M, Cattaneo A, Burrone OR (2004). Effects of intrabodies specific for rotavirus NSP5 during the virus replicative cycle. J Gen Virol.

[12] Mazuc E, Guglielmi L, Bec N, Parez V, Hahn CS, Mollevi C (2014). In-cell intrabody selection from a diverse human library identifies C12orf4 protein as a new player in rodent mast cell degranulation. PLoS ONE.

[13] Winter G, Griffiths AD, Hawkins RE, Hoogenboom HR (1994). Making antibodies by phage display technology. Annu Rev Immunol.

[14] Willats WG (2002). Phage display: practicalities and prospects. Plant Mol Biol.

[15] Huang JX, Bishop-Hurley SL, Cooper MA (2012). Development of anti-infectives using phage display: biological agents against bacteria, viruses, and parasites. Antimicrob Agents Chemother.

[16] Parray HA, Chiranjivi AK, Asthana S, Yadav N, Shrivastava T, Mani S (2020). Identification of an anti-SARS-CoV-2 receptor-binding domain-directed human monoclonal antibody from a naive semisynthetic library. J Biol Chem.

[17] Prins M, Lohuis D, Schots A, Goldbach R (2005). Phage display-selected single-chain antibodies confer high levels of resistance against Tomato spotted wilt virus. J Gen Virol.

[18] Deutscher SL (2010). Phage display in molecular imaging and diagnosis of cancer. Chem Rev.

[19] Wang Y, Gao S, Lv J, Lin Y, Zhou L, Han L (2019). Phage display technology and its applications in cancer immunotherapy. Anticancer Agents Med Chem.

[20] Zhou C, Kang J, Wang X, Wei W, Jiang W (2015). Phage display screening identifies a novel peptide to suppress ovarian cancer cells in vitro and in vivo in mouse models. BMC Cancer.

[21] Ferreira D, Silva AP, Nobrega FL, Martins IM, Barbosa-Matos C, Granja S (2019). Rational identification of a colorectal cancer targeting peptide through phage display. Sci Rep.

[22] Xie J, Yea K, Zhang H, Moldt B, He L, Zhu J (2014). Prevention of cell death by antibodies selected from intracellular combinatorial libraries. Chem Biol.

[23] McCafferty J (2014). Phenotypic directed antibody selection. Chem Biol.

[24] Wu DT, Seita Y, Zhang X, Lu CW, Roth MJ (2012). Antibody-directed lentiviral gene transduction for live-cell monitoring and selection of human iPS and hES cells. PLoS ONE.

[25] Huang SW, Tai CH, Fonville JM, Lin CH, Wang SM, Liu CC (2015). Mapping enterovirus A71 antigenic determinants from viral evolution. J Virol.

[26] Huang SW, Wang YF, Yu CK, Su IJ, Wang JR (2012). Mutations in VP2 and VP1 capsid proteins increase infectivity and mouse lethality of enterovirus 71 by virus binding and RNA accumulation enhancement. Virology.

[27] Andris-Widhopf J, Steinberger P, Fuller R, Rader C, Barbas CF, 3rd. Generation of human scFv antibody libraries: PCR amplification and assembly of light- and heavy-chain coding sequences. Cold Spring Harbor protocols. 2011;2011(9). 10.1101/pdb.prot065573 21880816

[28] Organization WH. WHO manual on animal influenza diagnosis and surveillance. World Health Organization, Geneva, Switzerland.; 2002.

[29] Dimitrov JD, Planchais C, Roumenina LT, Vassilev TL, Kaveri SV, Lacroix-Desmazes S (2013). Antibody polyreactivity in health and disease: statu variabilis. J Immunol.

[30] Lin JY, Li ML, Huang PN, Chien KY, Horng JT, Shih SR (2008). Heterogeneous nuclear ribonuclear protein K interacts with the enterovirus 71 5’ untranslated region and participates in virus replication. J Gen Virol.

[31] Shih SR, Stollar V, Li ML (2011). Host factors in enterovirus 71 replication. J Virol.

[32] Kuo RL, Lin YH, Wang RY, Hsu CW, Chiu YT, Huang HI (2015). Proteomics analysis of EV71-infected cells reveals the involvement of host protein NEDD4L in EV71 replication. J Proteome Res.

[33] Diaz-Ramos A, Roig-Borrellas A, Garcia-Melero A, Lopez-Alemany R (2012). alpha-Enolase, a multifunctional protein: its role on pathophysiological situations. J Biomed Biotechnol.

[34] Kishimoto N, Yamamoto K, Iga N, Kirihara C, Abe T, Takamune N (2020). Alpha-enolase in viral target cells suppresses the human immunodeficiency virus type 1 integration. Retrovirology.

[35] Kishimoto N, Yamamoto K, Abe T, Yasuoka N, Takamune N, Misumi S (2021). Glucose-dependent aerobic glycolysis contributes to recruiting viral components into HIV-1 particles to maintain infectivity. Biochem Biophys Res Commun.

[36] Leong WF, Chow VT (2006). Transcriptomic and proteomic analyses of rhabdomyosarcoma cells reveal differential cellular gene expression in response to enterovirus 71 infection. Cell Microbiol.

[37] Chan SY, Sam IC, Lai JK, Chan YF (2015). Cellular proteome alterations in response to enterovirus 71 and coxsackievirus A16 infections in neuronal and intestinal cell lines. J Proteomics.

[38] Too IHK, Bonne I, Tan EL, Chu JJH, Alonso S (2018). Prohibitin plays a critical role in Enterovirus 71 neuropathogenesis. PLoS Pathog.

[39] Xiang-Chun D, Xiao-Qing Y, Ting-Ting Y, Zhen-Hui L, Xiao-Yan L, Xia L (2018). Alpha-enolase regulates hepatitis B virus replication through suppression of the interferon signalling pathway. J Viral Hepat.

[40] Miles LA, Dahlberg CM, Plescia J, Felez J, Kato K, Plow EF (1991). Role of cell-surface lysines in plasminogen binding to cells: identification of alpha-enolase as a candidate plasminogen receptor. Biochemistry.

[41] Dudani AK, Cummings C, Hashemi S, Ganz PR (1993). Isolation of a novel 45 kDa plasminogen receptor from human endothelial cells. Thromb Res.

[42] Aaronson RM, Graven KK, Tucci M, McDonald RJ, Farber HW (1995). Non-neuronal enolase is an endothelial hypoxic stress protein. J Biol Chem.

[43] Subramanian A, Miller DM (2000). Structural analysis of alpha-enolase. Mapping the functional domains involved in down-regulation of the c-myc protooncogene. J Biol Chem.

[44] Niklinski J, Furman M (1995). Clinical tumour markers in lung cancer. Eur J Cancer Prev.

[45] Ledermann JA (1994). Serum neurone-specific enolase and other neuroendocrine markers in lung cancer. Eur J Cancer.

[46] Eriksson B, Oberg K, Stridsberg M (2000). Tumor markers in neuroendocrine tumors. Digestion.

[47] Takashima M, Kuramitsu Y, Yokoyama Y, Iizuka N, Fujimoto M, Nishisaka T (2005). Overexpression of alpha enolase in hepatitis C virus-related hepatocellular carcinoma: association with tumor progression as determined by proteomic analysis. Proteomics.

[48] Ye Y, Kuhn C, Kosters M, Arnold GJ, Ishikawa-Ankerhold H, Schulz C (2019). Anti alpha-enolase antibody is a novel autoimmune biomarker for unexplained recurrent miscarriages. EBioMedicine.

[49] Akisawa N, Maeda T, Iwasaki S, Onishi S (1997). Identification of an autoantibody against alpha-enolase in primary biliary cirrhosis. J Hepatol.

[50] Saulot V, Vittecoq O, Charlionet R, Fardellone P, Lange C, Marvin L (2002). Presence of autoantibodies to the glycolytic enzyme alpha-enolase in sera from patients with early rheumatoid arthritis. Arthritis Rheum.

[51] Bae S, Kim H, Lee N, Won C, Kim HR, Hwang YI (2012). alpha-Enolase expressed on the surfaces of monocytes and macrophages induces robust synovial inflammation in rheumatoid arthritis. J Immunol.

[52] Shih NY, Lai HL, Chang GC, Lin HC, Wu YC, Liu JM (2010). Anti-alpha-enolase autoantibodies are down-regulated in advanced cancer patients. Jpn J Clin Oncol.

[53] Zhang L, Wang H, Dong X (2018). Diagnostic value of alpha-enolase expression and serum alpha-enolase autoantibody levels in lung cancer. J Bras Pneumol.

[54] Dai L, Qu Y, Li J, Wang X, Wang K, Wang P (2017). Serological proteome analysis approach-based identification of ENO1 as a tumor-associated antigen and its autoantibody could enhance the sensitivity of CEA and CYFRA 21–1 in the detection of non-small cell lung cancer. Oncotarget.

[55] Han L, Li K, Jin C, Wang J, Li Q, Zhang Q (2017). Human enterovirus 71 protein interaction network prompts antiviral drug repositioning. Sci Rep.

[56] Zou P, Yang Y, Xu X, Liu B, Mei F, You J (2018). Silencing of vacuolar ATPase c subunit ATP6V0C inhibits the invasion of prostate cancer cells through a LASS2/TMSG1-independent manner. Oncol Rep.

[57] Sennoune SR, Luo D, Martinez-Zaguilan R (2004). Plasmalemmal vacuolar-type H+-ATPase in cancer biology. Cell Biochem Biophys.

[58] Peng B, Huang X, Nakayasu ES, Petersen JR, Qiu S, Almeida IC (2013). Using immunoproteomics to identify alpha-enolase as an autoantigen in liver fibrosis. J Proteome Res.

[59] Lu BR, Brindley SM, Tucker RM, Lambert CL, Mack CL (2010). alpha-enolase autoantibodies cross-reactive to viral proteins in a mouse model of biliary atresia. Gastroenterology.

[60] Anthony RM, Wermeling F, Karlsson MC, Ravetch JV (2008). Identification of a receptor required for the anti-inflammatory activity of IVIG. Proc Natl Acad Sci U S A.

[61] Han JF, Cao RY, Deng YQ, Tian X, Jiang T, Qin ED (2011). Antibody dependent enhancement infection of enterovirus 71 in vitro and in vivo. Virol J.

[62] Chiramel AI, Brady NR, Bartenschlager R (2013). Divergent roles of autophagy in virus infection. Cells.

[63] Huang SC, Chang CL, Wang PS, Tsai Y, Liu HS (2009). Enterovirus 71-induced autophagy detected in vitro and in vivo promotes viral replication. J Med Virol.

[64] Xi X, Zhang X, Wang B, Wang T, Wang J, Huang H (2013). The interplays between autophagy and apoptosis induced by enterovirus 71. PLoS ONE.

[65] Shu X, Cao KY, Liu HQ, Yu L, Sun LX, Yang ZH (2021). Alpha-enolase (ENO1), identified as an antigen to monoclonal antibody 12C7, promotes the self-renewal and malignant phenotype of lung cancer stem cells by AMPK/mTOR pathway. Stem Cell Res Ther.

[66] Yao F, Zhang M, Chen L (2016). 5’-Monophosphate-activated protein kinase (AMPK) improves autophagic activity in diabetes and diabetic complications. Acta Pharm Sin B.

[67] Didiasova M, Schaefer L, Wygrecka M (2019). When place matters: shuttling of enolase-1 across cellular compartments. Front Cell Dev Biol.

[68] Baixauli F, Lopez-Otin C, Mittelbrunn M (2014). Exosomes and autophagy: coordinated mechanisms for the maintenance of cellular fitness. Front Immunol.

[69] Ojha CR, Lapierre J, Rodriguez M, Dever SM, Zadeh MA, DeMarino C (2017). Interplay between autophagy, exosomes and HIV-1 associated neurological disorders: new insights for diagnosis and therapeutic applications. Viruses.

[70] Salimi L, Akbari A, Jabbari N, Mojarad B, Vahhabi A, Szafert S (2020). Synergies in exosomes and autophagy pathways for cellular homeostasis and metastasis of tumor cells. Cell Biosci.

[71] Cappello P, Principe M, Bulfamante S, Novelli F (2017). Alpha-Enolase (ENO1), a potential target in novel immunotherapies. Front Biosci (Landmark Ed).

